# Acute Flaccid Paralysis and West Nile Virus Infection

**DOI:** 10.3201/eid0907.030129

**Published:** 2003-07

**Authors:** James J. Sejvar, A. Arturo Leis, Dobrivoje S. Stokic, Jay A. Van Gerpen, Anthony A. Marfin, Risa Webb, Maryam B. Haddad, Bruce C. Tierney, Sally A. Slavinski, Jo Lynn Polk, Victor Dostrow, Michael Winkelmann, Lyle R. Petersen

**Affiliations:** * Centers for Disease Control and Prevention, Atlanta, Georgia, USA; † Methodist Rehabilitation Center, Jackson, Mississippi, USA; ‡ Ochsner Clinic, New Orleans, Louisiana, USA; § Mississippi State Department of Health, Jackson, Mississippi, USA

**Keywords:** West Nile virus, poliomyelitis, spinal cord, electromyography, muscle, weakness, Guillain-Barré syndrome, research

## Abstract

Acute weakness associated with West Nile virus (WNV) infection has previously been attributed to a peripheral demyelinating process (Guillain-Barré syndrome); however, the exact etiology of this acute flaccid paralysis has not been systematically assessed. To thoroughly describe the clinical, laboratory, and electrodiagnostic features of this paralysis syndrome, we evaluated acute flaccid paralysis that developed in seven patients in the setting of acute WNV infection, consecutively identified in four hospitals in St. Tammany Parish and New Orleans, Louisiana, and Jackson, Mississippi. All patients had acute onset of asymmetric weakness and areflexia but no sensory abnormalities. Clinical and electrodiagnostic data suggested the involvement of spinal anterior horn cells, resulting in a poliomyelitis-like syndrome. In areas in which transmission is occurring, WNV infection should be considered in patients with acute flaccid paralysis. Recognition that such weakness may be of spinal origin may prevent inappropriate treatment and diagnostic testing.

Most human infections with West Nile virus (WNV), a flavivirus within the Japanese encephalitis virus antigenic complex, are clinically inapparent (*1*,*2*). Mild febrile illness develops in approximately 1 in 5 infected persons; more severe neurologic disease, mostly meningitis or encephalitis, occurs in and 1 in 150 (*1*–*4*). Less frequently, acute WNV infection has been associated with acute flaccid paralysis, which has been attributed to Guillain-Barré syndrome, motor axonopathy, or axonal polyneuropathy (*4*–*6*). However, these reports describe clinical and laboratory features that seem inconsistent with such diagnoses, and the exact cause of acute flaccid paralysis has not been thoroughly assessed with rigorous electrophysiologic, laboratory, and neuroimaging data. Brief descriptions of six patients have suggested that this flaccid paralysis is due to anterior horn cell involvement with a resultant poliomyelitis-like syndrome (*7*–*9*). Because understanding the clinical characteristics and underlying etiology of WNV-induced acute flaccid paralysis is critical for therapeutic decisions as well as prognosis, we describe the detailed clinical, laboratory, and electrophysiologic findings from these six patients and from one additional patient.

## Patients and Methods

Seven patients were detected through WNV surveillance conducted by the Mississippi Department of Health and the Louisiana Office of Public Health. For each patient, a standardized questionnaire, including demographics, medical history, initial signs and symptoms, risk factors, and treatment, was completed; a standardized neurologic examination was performed by a single neurologist (JJS). Electrodiagnostic studies were performed by neurologists (AAL and JAVG) specializing in electrodiagnostic medicine.

Cerebrospinal fluid (CSF) and acute- or convalescent-phase serum specimens (or both) from each patient were tested for antibody to WNV by immunoglobulin (Ig) M antibody-capture enzyme immunoassay (*10*) or plaque reduction neutralization assay (*11*). The initial specimen for one patient (patient 5, Table 1) was tested with a slightly modified IgM antibody assay at a commercial laboratory (*12*). IgM assays were considered positive if the optical density ratio of the patient and negative control samples (P/N ratio) was greater than three. For patient samples, a P/N ratio for WNV at least three times that for St. Louis encephalitis virus indicated WNV infection (*13*). A plaque reduction neutralization test result of at least 10 was considered positive.

**Table 1:** Serologic results for West Nile virus (WNV)–specific antibodies in patients with acute flaccid paralysis associated with acute WNV infection^a^

				IgM-capture enzyme immunoassay		Plaque reduction neutralization assay	
Case	Onset	Collection	Sample	SLEV	WNV	SLEV	WNV
1	6/24	7/12	Serum	3.5	22.3	320	5,120
							
2	7/12	7/16	Serum	8.0	22.7	80	1,280
							
3	7/26	8/1	Serum	2.79	24.9	<10	640
							
4	7/29	8/3	Serum	1.1	14.1	<10	80
4	7/29	8/3	CSF	3.3	39.2		
4	7/29	8/13	Serum	4.4	23.5	40	2,560
							
5	8/11	8/15	Serum		2.02		
5	8/11	8/29	Serum	3.4	25.7		
							
6	8/13	8/16	CSF	6.1	23.8		
6	8/13	8/16	Serum	1.0	5.7	<10	40
							
7	9/1	10/24	Serum	2.8	10.6	10	320
7	9/1	9/6	CSF	Not performed	7.4		

^a^IgM, immunoglobulin M; SLEV, Saint Louis encephalitis virus; CSF, cerebrospinal fluid.

All seven patients had serologic evidence of WNV infection (Table 1). On the basis of serologic data, three of the patients were classified as confirmed case-patients (patients 4, 6, and 7) and four as probable case-patients (patients 1–3, 5), according to the national case definition (*14*).

### Case 1

On July 1, 2002, a previously healthy, 56-year-old, male Mississippi resident was hospitalized with a 1-week history of fever, chills, night sweats, myalgias, and acute encephalopathy. Neurologic examination showed profound weakness in both arms, asymmetric weakness in the legs with a right foot drop, and acute respiratory distress (Table 2). Sensory test results were normal. Although computed tomography (CT) and magnetic resonance imaging (MRI) of the brain showed normal results, heparin was administered for suspected evolving stroke. Admission laboratory values (Table 3) showed serum leukocytosis, and cerebrospinal fluid (CSF) obtained on day 3 showed elevated protein. By day 8, Guillain-Barré syndrome was suspected, and intravenous immunoglobulin (IVIG) was administered. Electrodiagnostic studies performed that day were interpreted as showing a proximal neuropathy or myopathy. A deltoid muscle biopsy for suspected inflammatory myopathy showed mild type 2 fiber atrophy but no obvious necrosis or marked inflammatory response. On day 30, the patient was transferred for rehabilitation with flaccid, areflexic paralysis in the right leg and variable weakness and diminished reflexes in all other limbs. Neck flexors were normal in strength, and sensation was preserved in all limbs; loss of bladder function was evident. MRI of the cervical spine showed normal results. Electrodiagnostic studies showed widespread but variable denervation, reduced compound muscle action potentials (CMAPs), and normal sensory nerve action potentials (SNAPs), consistent with a severe, asymmetric process affecting anterior horn cells or motor axons. Myopathy, demyelinating polyneuropathy, and diffuse axonal polyneuropathy were not evident.

**Table 2:** Initial clinical signs and symptoms in patients with acute flaccid paralysis associated with acute West Nile virus infection

Case no.	Fever (>38.5°C)	Headache	Nuchal rigidity	Altered mental status	Tremor	Distribution of weakness^a^
1	+	+	+	+	+	Upper and lower limbs, R > L
						
2	+	+	-	+	-	Upper and lower limbs, R > L
						
3	+	-	-	-	+	Lower limbs, R > L
						
4	+	+	+	+	+	R upper limb
						
5	-	+	-	-	-	R upper limb
						
6	+	+	-	-	+	Lower limbs, R > L
						
7	+	+	+	-	-	Upper and lower limbs, L > R; bulbar muscles

^a^R, right; L, left.

**Table 3:** Initial laboratory findings in patients with acute flaccid paralysis associated with acute West Nile virus infection^a^

Case no.	Leukocytes (x10^3^/mm^3^)	Hematocrit (%)	CSF WBC (/mm^3^)	CSF RBC (/mm^3^)	CSF protein (mg/dL)	CSF glucose (mg/dL)
1	17.6	38.0	3	1,778	89	54
						
2	3.6	38.2	2,600	87	204	99
						
3	11.8	44.4	140	40	234	74
						
4	9.5	37.8	143	4	116	119
						
5	7.9	45.6	Not performed	Not performed	Not performed	Not performed
						
6	13.0	45.4	329	7	75	66
						
7	10.3	Not performed	182	9	37	79

^a^CSF, cerebrospinal fluid; WBC, leukocyte count; RBC, erythrocyte count.

### Case 2

On July 15, 2002, a 57-year-old male Mississippi resident with a remote history of prostate cancer and glucose intolerance was hospitalized with a 3-day history of fever, chills, nausea, vomiting, and headache. Neurologic examination showed encephalopathy and asymmetric weakness in all limbs (Table 2). Results of a brain MRI weree normal. Admission laboratory studies showed a CSF pleocytosis with elevated protein (Table 3). On day 5, acute respiratory distress developed, and the patient required mechanical ventilation. Upon extubation 2 weeks later, the patient had continued extremity weakness and was aspirating fluids; a modified barium swallow study showed oropharyngeal dysphagia. Upon transfer to a rehabilitation center on day 30, the patient had asymmetric weakness in the legs and right arm and moderate weakness in neck flexors and facial muscles. Hypotonia and areflexia were noted in all limbs. Sensation was slightly diminished to vibration and proprioception in toes bilaterally but preserved to light touch, pinprick, and temperature. Sensory testing was normal in the upper limbs. Urinary incontinence was noted. Electrodiagnostic studies showed widespread denervation, reduced CMAP amplitudes in all nerves of the lower limbs and right upper limb, and normal SNAP responses, consistent with a severe, asymmetric process affecting anterior horn cells or motor axons. Myopathy, demyelinating polyneuropathy, and diffuse axonal polyneuropathy were not apparent.

### Case 3

On July 24, 2002, a low-grade fever, nausea, and vomiting, followed by shaking chills and sweats, developed in a 56-year-old male Louisiana resident with a history of hypertension and coronary artery disease. The next day, asymmetric weakness developed in the lower extremities, with no pain or numbness. Upper extremities were normal. No bowel or bladder dysfunction was present. The patient was hospitalized on July 29, and neurologic examination showed a flaccid, areflexic right lower extremity and a weak left lower extremity with diminished reflexes. Results of strength and reflex testing of the upper extremities were normal. Sensory examination results were normal except for a mild decrease in sensitivity to pinprick, temperature, touch, and vibration in a stocking-and-glove distribution (i.e., distal arms and legs). A coarse bilateral upper extremity action tremor was noted. The patient had no headache, neck stiffness, or alteration of mental status (Table 2). Admission laboratory values showed leukocytosis and CSF pleocytosis (Table 3). Results of other diagnostic tests were unremarkable. Postviral demyelination syndrome and viral-induced polyradicultis were considered, and IVIG**,** dexamethasone, and antibacterial and antiviral medications were administered without patient improvement. On day 15, the patient was discharged to a skilled nursing facility for rehabilitation.

MRI of the cervical, thoracic, and lumbosacral spine obtained during rehabilitation was notable for showing mild cervical and lumbosacral spinal stenosis and foraminal restrictions from C3 through C7 and homogeneous enhancement of the nerve roots of the cauda equina consistent with meningitis. Electrodiagnostic studies showed denervation in thoracic and lumbosacral myotomes, with no muscle activation in the right leg and reduced muscle activation in the left leg. CMAPs in the right leg were absent; SNAPs were normal. Electrodiagnostic findings suggested a severe, asymmetric process affecting anterior horn cells or motor axons. Diffuse axonal polyneuropathy was not evident, despite a slight sensory loss in the distal extremities.

### Case 4

On August 2, 2002, fever, headache, and neck stiffness developed in a 69-year-old female Louisiana resident with a history of diabetes and degenerative disc disease; the next day acute weakness occurred in the right arm without pain, numbness, or paresthesias. She was hospitalized on August 4. On admission, physical examination documented fever, vomiting, encephalopathy, nuchal rigidity, and a bilateral rash on the lower extremities. Neurologic examination displayed a flaccid and areflexic right arm. Her legs and left arm exhibited normal strength, reflexes, and coordination, with normal sensation in all limbs. A coarse tremor was noted in the chin, left arm, and legs (Table 2). Laboratory findings included CSF pleocytosis (Table 3). Differential diagnoses included meningoencephalitis with associated motor polyradiculopathy and monoplegia secondary to stroke. The patient was treated with antibacterial and antiviral medications. Results of CT and MRI of the brain were normal. MRI of the cervical spine showed multilevel degenerative disc disease. The patient remained lethargic until day 13, when mental status abruptly improved; right arm weakness persisted. On day 19, she was transferred to a rehabilitation facility. Electrodiagnostic studies showed absent CMAPs and profound denervation with no voluntary activation in muscles of the right arm. Scattered denervation was also seen in the other three limbs. SNAPs had borderline amplitudes and conduction velocities bilaterally. The results were most consistent with a severe, asymmetric process affecting anterior horn cells or motor axons. The patient was subsequently transferred back to intensive care because her respiratory function was deteriorating, but she was not intubated. After Guillain-Barré syndrome was diagnosed, she was started on IVIG but had no improvement in weakness.

### Case 5

On August 11, 2002, severe nausea, vomiting, headache, and diarrhea in the absence of fever developed in a 50-year-old male Mississippi resident with a history of alcohol abuse; the next day, progressive right arm weakness developed. He was hospitalized on August 14. Neurologic examination on admission showed flaccid paralysis of the right arm and mild weakness of the right leg, with normal sensation in all limbs (Table 2). Laboratory values are shown in Table 3; a lumbar puncture was not performed. Acute stroke was diagnosed, and the patient was treated with heparin. Mental status changes, dysarthria, and dysphagia subsequently developed but resolved. Upon transfer to a rehabilitation center on day 12, the patient had paralysis and areflexia limited to the right arm, with normal sensation and diffuse tremor in all limbs. Brain MRI results were normal; cervical spine MRI displayed mild multilevel foraminal stenosis on the left. Electrodiagnostic studies showed markedly reduced motor responses in the right arm with normal sensory responses, consistent with a severe asymmetric process affecting anterior horn cells.

### Case 6

On August 16, 2002, a 46-year-old male Louisiana resident with a history of coronary artery disease was hospitalized with fever, headache, fatigue, and leg weakness of 3 days’ duration. He reported no nuchal rigidity or mental status changes, although family members described him as intermittently confused. Neurologic examination showed a plegic and areflexic right leg and mild left leg weakness; sensation was intact throughout. A bilateral tremor of the upper extremities and jaw was noted (Table 2). Laboratory abnormalities included a CSF pleocytosis (Table 3). He was diagnosed with Guillain-Barré syndrome and started on IVIG. Brain CT and MRI results were normal. Results of an enhanced MRI of the spine suggested meningitis involving the conus medullaris and cauda equina. Electrodiagnostic studies performed on day 4 demonstrated early denervation and absent activation in muscles of the right leg and reduced activation of muscles in the right arm. CMAPs and SNAPs in the right arm and leg were normal. These findings were consistent with a severe, asymmetric process affecting anterior horn cells or motor axons. He was transferred to a rehabilitation facility on day 6 with no improvement of weakness.

### Case 7

On September 1, 2002, a previously healthy, 39-year-old male Louisiana resident had onset of fever, headache, and nuchal rigidity followed the next day by dysphagia and bilateral arm and leg weakness that was worse on the left. He was hospitalized on September 6 for acute respiratory failure and intubated. Neurologic examination showed normal cognition, asymmetric flaccid paralysis of the left arm and leg with absent reflexes, hyporeflexic weakness of the right arm and leg, and weakness of bulbar muscles (Table 2). A partial supranuclear gaze palsy, cogwheel rigidity, and bilateral Babinski signs were also evident. Admission laboratory findings showed peripheral leukocytosis and CSF pleocytosis (Table 3). Brain MRI showed increased T2 signal in the periaqueductal gray matter, substantia nigra, and trigeminal motor nuclei. Electrodiagnostic studies performed on day 15 showed diffuse denervation in all myotomes, reduced CMAPs (worse on the left), and preserved SNAPs. On day 25, he was transferred to a long-term care facility with no improvement of limb weakness.

## Discussion

The clinical and electrodiagnostic findings in these patients with WNV infection suggest involvement of spinal cord gray matter, specifically anterior horn cells, and a resulting acute poliomyelitis-like syndrome. All patients exhibited features typical for polio, including acute flaccid paralysis without paresthesias or sensory loss, marked asymmetric weakness, diminished or absent deep tendon reflexes in the affected limbs, and weakness that developed during an acute infectious process. Other typical features of poliomyelitis included CSF pleocytosis in five of six patients with CSF examination, acute respiratory distress in four, and acute changes in bowel or bladder function in two. In addition, electrodiagnostic findings showed asymmetric muscle denervation, reduced CMAPs, and preserved SNAPs. No patients had evidence of demyelinating polyneuropathy or myopathy. The absence of new sensory abnormalities localizes the disease process to the anterior horn cells or motor axons. Although muscle denervation and reduced CMAP amplitudes do not distinguish loss of anterior horn cells from loss of motor axons (*15*), these patients’ clinical features can be explained only by anterior horn cell disease, since no known infectious processes limited to motor axons produce widespread, asymmetric paralysis without sensory involvement. While MRI signal abnormalities in the anterior spinal cord have been noted in patients with poliomyelitis (*16*,*17*), these findings are inconsistent (*18*,*19*), and the absence of such changes in our four patients in which imaging was performed does not preclude a diagnosis of a poliomyelitis-like syndrome.

Since immunization has eradicated wild-type poliovirus from the developed world, most cases of paralytic polio-like conditions in the United States have been linked to other RNA viruses, including echoviruses, enteroviruses, and coxsackieviruses (*20*). Case reports have documented a poliomyelitis-type syndrome associated with other flaviviruses (*21*–*23*), as well as anterior myelitis associated with WNV infection (*24*).

The assertion that WNV infection involves anterior horn cells and causes a polio-type syndrome has a pathologic basis. The neuropathology of experimental WNV infection in monkeys was most pronounced in the cerebellum, medulla, and the cervical and lumbar regions of the spinal cord (*25*). Anterior horn cells showed degeneration and neuronal cell death; conversely, no changes were seen in the oligodendroglia or peripheral nerves. Similarly, WNV-infected horses displayed multifocal polioencephalomyelitis, with involvement of the ventral and lateral horns of the thoracic and lumbar spinal cord (*26*,*27*). WNV antigen was mainly localized within the gray matter of the spinal cord, with no lesions apparent in peripheral nerves or ganglia. In WNV-infected birds, lesions and viral antigen were most prominent in the cerebellum and the gray matter of the spinal cord (*28*).

Previous case studies have attributed WNV-associated acute flaccid paralysis to Guillain-Barré syndrome, motor axonopathy, or severe axonal polyneuropathy (*4*–*6*). The clinical signs and symptoms and electrodiagnostic findings reported in those cases, and those described here, are most consistent with a polio-like condition, and would be atypical for Guillain-Barré syndrome or other peripheral nerve disorders. Although acute poliomyelitis and polio-like conditions may occasionally simulate Guillain-Barré syndrome (*29*), our cases had several clinical, laboratory, and electrodiagnostic features that differed from typical Guillain-Barré syndrome (*30**–**32*; Table 4).

**Table 4:** Clinical characteristics of patients with West Nile virus–associated acute flaccid paralysis compared with patients with typical Guillain-Barré syndrome (25–27)^a^

Characteristic	West Nile virus–associated flaccid paralysis	Guillain-Barré syndrome
Timing of onset	Acute phase of infection	1–8 weeks after acute infection
Fever and leukocytosis	Present	Absent
Weakness distribution	Asymmetric; occasional monoplegia	Generally symmetric; proximal and distal muscles
Sensory symptoms	Absence of numbness, paresthesias, or sensory loss; occasional myalgias	Painful distal paresthesias and sensory loss
Bowel/bladder involvement	Often present	Rare
Concurrent encephalopathy	Often present	Absent
CSF profile	Pleocytosis and elevated protein	No pleocytosis; elevated protein (albuminocytologic dissociation)
Electrodiagnostic features	Anterior horn cell/motor axon: reduced/absent CMAPs, preserved SNAPs; asymmetric denervation	Demyelination: marked slowing of conduction velocity; conduction block, temporal dispersion; reduced SNAPs

^a^CSF, cerebrospinal fluid; CMAPs, compound muscle action potentials; SNAPSs, sensory nerve action potentials.

In Guillain-Barré syndrome, electrodiagnostic findings generally suggest peripheral nerve demyelination or, less commonly, a combined demyelinating and axonal process (*30*,*31*). The cases reported here displayed reduced or absent CMAPs with preserved SNAPs, no evidence of demyelination, a neurogenic pattern of recruitment, and widespread denervation; combined with the clinical picture of an asymmetric paralysis, these findings are typical for a polio-like condition and uncommon for Guillain-Barré syndrome. A pure axonal variant of Guillain-Barré syndrome has been described (*33*) and may be confused with poliomyelitis and polio-like conditions; however, such cases are generally characterized by distally prominent weakness and show subclinical sensory nerve involvement on electrodiagnostic testing. Thus, in the context of WNV infection, electrodiagnostic studies previously interpreted as motor axonal polyneuropathy or motor axonopathy without sensory nerve involvement (*4*–*6*) are more suggestive of anterior horn cell loss than of Guillain-Barré syndrome.

Three of the seven patients had acute flaccid paralysis without other findings, suggestive of severe central nervous system involvement caused by WNV infection. Physicians should suspect WNV infection in patients from areas where WNV is being transmitted and who have acute, painless, asymmetric weakness, even if unaccompanied by fever or apparent meningoencephalitis. Diagnostic studies should include testing for WNV-specific IgM antibody in CSF or acute- and convalescent-phase serum samples. In patients from such areas who have acute flaccid paralysis, CSF analysis, thorough electrodiagnostic studies, and spinal imaging should be considered before initiating diagnostic evaluations or therapies directed at Guillain-Barré syndrome, stroke, inflammatory myopathies, or other peripheral inflammatory processes. These therapies are ineffective for polio-like syndromes and can produce serious sequelae (*34*–*37*).

Continued surveillance and investigation of WNV-infected patients are needed to fully define the scope of clinical illness and determine the incidence of acute flaccid paralysis. In addition to assessing clinical outcome, the identification of risk factors and the pathologic confirmation of anterior horn cell involvement in patients with WNV-associated acute flaccid paralysis remain important public health goals.
